# College Members whose deaths were reported at Council meetings between October 2019 and October 2020

**DOI:** 10.1192/bjb.2020.134

**Published:** 2021-02

**Bandali,** Sameer Kamaluddin

**Member**

**Beckett,** Henry Dale

**Member**

**Bethell,** Maxwell Slingsby

**Fellow ~ Over 40 Years member**

**Bhaumik,** Sabyasachi

**Honorary Fellow**

**Carroll,** Bernard James

**Fellow ~ Over 40 Years member**

**Fleminger,** John Jack

**Fellow ~ Over 40 Years member**

**Gosselin,** Jean-Yves

**Fellow ~ Over 40 Years member**

**Heine,** Bernard Edmund

**Fellow ~ Over 40 Years member**

**Holden,** Hyla Montgomery

**Member ~ Over 40 Years member**

**Kanakaratnam,** Gunaseelan

**Fellow ~ Over 40 Years member**

**Kanjilal,** Gopal Chandra

**Fellow ~ Over 40 Years member**

**Kolakowska,** Tamara

**Retired**

**Member ~ Over 40 Years member**

**May,** Kathleen Mackenzie

**Member ~ Over 40 Years member**

**Mirza,** Yousuf Kamal

**Consultant**

**Member**

**Scott,** John Clelland

**Fellow ~ Over 40 Years member**

**Sirag,** Ahmed Osman

**Retired**

**Fellow**

**Wat,** Hong-Yun Karen **Member**

**Alexander,** David A

**Honorary Fellow**

**Alexander,** Eric Richardson

**Member ~ Over 40 Years member**

**Allan,** Frances

**Member ~ Over 40 Years member**

**Beauchemin,** Barbara Susannah Seymour

**Affiliate**

**Burkitt,** Eric Aylmer

**Fellow ~ Over 40 Years member**

**Dadds,** Violet Elsie

**Member**

**Davies,** Roy James

**Fellow ~ Over 40 Years member**

**de Mowbray,** Michael Stuart

**Member**

**Denham,** Maureen Milburn

**Member ~ Over 40 Years member**

**Engelhardt,** Wolfram Detlev Achim

**Member**

**Fenton,** Thomas William

**Fellow ~ Over 40 Years member**

**Forrest,** Alastair John

**Fellow ~ Over 40 Years member**

**Gallagher,** Elizabeth Gibb

**Member ~ Over 40 Years member**

**Garry,** John William

**Fellow ~ Over 40 Years member**

**Gillham,** Adrian Bayley

**Fellow**

**Gray,** Anne Margaret

**Fellow ~ Over 40 Years member**

**Grimshaw,** John Stuart

**Fellow ~ Over 40 Years member**

**Hamour,** Mohamed Abdelaal

**Member**

**Hersov,** Lionel Abraham

**Fellow ~ Over 40 Years member**

**Iskander,** Trevor Nagib

**Member ~ Over 40 Years member**

**Kelleher,** F Joseph

**Member ~ Over 40 Years member**

**Knox,** Stafford Joseph

**Fellow ~ Over 40 Years member**

**Leslie,** Nasnaranpattiyage Don George

**Member ~ Over 40 Years member**

**Macleod,** Iain Roderic William

**Member**

**Masih,** Harnek

**Member**

**Matthews,** Peter Charles

**Member ~ Over 40 Years member**

**McNeill,** Desmond Lorne Marcus

**Fellow ~ Over 40 Years member**

**Nasser,** Zeinab Abdel-Aziz Ibrahim

**Member**

**Rogers,** Paul Haydon

**Fellow**

**Ryle,** Anthony

**Fellow ~ Over 40 Years member**

**Todes,** Cecil Jacob

**Fellow ~ Over 40 Years member**

**Youssef,** Hanafy Ahmed Mahmoud

**Fellow ~ Over 40 Years member**

**Arie,** Thomas Harry David

**Honorary Fellow**

**Pant,** Anshuman

**Member**

**Smith,** Eileen Dorothy

**Fellow ~ Over 40 Years member**

**White,** Daniel Paul

**Member**

**Chan,** Chee Hung

**Member**

**Coia,** Denise Assunda

**Honorary Fellow**

**Duddle,** Constance May

**Fellow ~ Over 40 Years member**

**Dunlop,** Joyce Lilian

**Fellow ~ Over 40 Years member**

**Hickling,** Frederick W

**Fellow ~ Over 40 Years member**

**Hilary-Jones,** Evan Peter

**Fellow**

**Jones,** David Alun

**Fellow**

**McGovern,** Gerald Patrick

**Fellow ~ Over 40 Years member**

**Pant,** Anshuman

**Member**

**Pathak,** Rudresh Kumar Dinanath

**Member**

**Smith,** Eileen Dorothy

**Fellow ~ Over 40 Years member**

**White,** Daniel Paul

**Member**

**D'Orban,** Paul T

Retired

**Fellow ~ Over 40 Years member**

**Hewland,** Helen Robyn

Retired

**Fellow ~ Over 40 Years member**

**Hughes,** John Samuel

Retired

**Fellow ~ Over 40 Years member**

**Imrie,** Alison Wendy

**Consultant**

**Member**

**Lader,** Malcolm Harold

**Emeritus Professor**

**Fellow ~ Over 40 Years member**

**McLaughlin,** Jo-Ann

Retired

**Member**

**Mubbashar,** Malik Hussain

**Consultant**

**Fellow ~ Over 40 Years member**

**Robinson,** John Richard

Retired

**Fellow ~ Over 40 Years member**

**Washbrook,** Reginald Alfred Hryhoruk

**Fellow ~ Over 40 Years member**

**Gillis,** Lynn

Retired

**Fellow**

**Kenyon,** Frank Edwin

Retired

**Fellow**

**Palmer**, Bob

**Fellow**

**Rana,** Mamoona

**West,** Donald

**Fellow**

**Brown,** Philip Morrison

**Fellow – North Division**

